# First person – Marte Molenaars and Bauke Schomakers

**DOI:** 10.1242/dmm.049009

**Published:** 2021-04-27

## Abstract

First Person is a series of interviews with the first authors of a selection of papers published in Disease Models & Mechanisms, helping early-career researchers promote themselves alongside their papers. Marte Molenaars and Bauke Schomakers are co-first authors on ‘Metabolomics and lipidomics in *Caenorhabditis elegans* using a single-sample preparation’, published in DMM. Marte conducted the research described in the article while a PhD student in the lab of Riekelt Houtkooper at Amsterdam UMC, Amsterdam, The Netherlands, and is now a postdoc in the lab of Richard Possemato at New York University School of Medicine, New York, NY, USA. During her PhD, she was investigating cellular metabolic pathways and how to use them to target ageing and age-related diseases, and currently she focuses on metabolic pathways in cancer cells as a postdoc. Bauke is a Research Analyst in the lab of Riekelt Houtkooper at Amsterdam UMC, Amsterdam, The Netherlands, investigating liquid chromatography–mass spectrometry-based omics methods for the comprehensive analysis of biological samples.


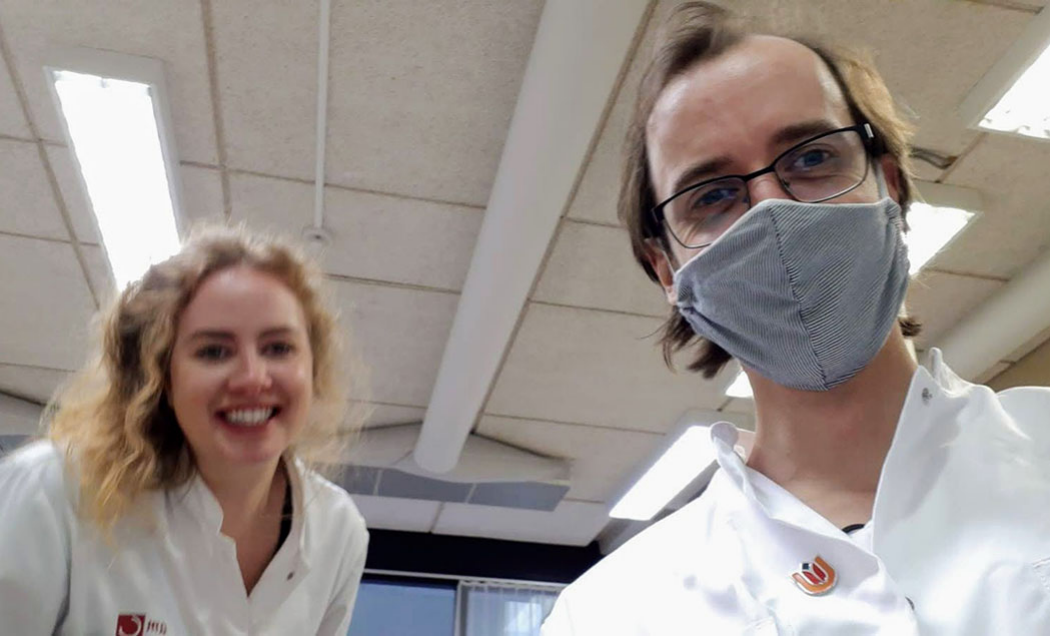


**Marte Molenaars and Bauke Schomakers**

**How would you explain the main findings of your paper to non-scientific family and friends?**

An important aspect of research related to nutrition and ageing is mapping the changes that take place in metabolism. For this, we use methods that can measure many intermediates of metabolism (metabolites) at once, a technique called metabolomics. The better we can map these metabolites, the better we are able to obtain a complete picture of the differences in metabolism.

In addition to metabolomics, which measures water-soluble metabolites such as amino acids and glucose, there are also fatty metabolites (lipids) that can be measured using a technique called lipidomics. Normally these methods each require their own experiment and workup. In our publication, we report that metabolomics and lipidomics can be performed with a single extraction protocol, which reduces preparation and turnaround time.

**What are the potential implications of these results for your field of research?**

We can now measure both water soluble metabolites as well as lipids from one single sample. This complete picture of metabolism from one sample can be extra useful in difficult-to-obtain material such as human biopsies. Besides reducing sample preparation and throughput time, it also requires a lower number of *C. elegans* cultures and results in a reduction in waste, without compromising metabolite identification.

**What are the main advantages and drawbacks of the model system you have used as it relates to the disease you are investigating?**

*C. elegans* has proven itself to be one of the most versatile model organisms for the elucidation of molecular pathways implicated in human diseases and ageing. *C. elegans* only lives 2-4 weeks, so we can do a lot of lifespan research in a short time. This kind of research in people would last up to 100 years. Interestingly, many important findings in fundamental biology and the medical field were first achieved in *C. elegans*, suggesting that molecular mechanisms and signalling pathways are conserved between mammals and worms. However, if you want to research processes in defined organs/tissues such as brain, blood, or internal organs, *C. elegans* might not be the optimal model organism.

As for our metabolomics/lipidomics method, while it was mostly validated in *C. elegans*, in the meantime we've already successfully used our method in cells, flies, fish, and human/mouse tissues and fluids.

“The better we understand and measure the differences in metabolism between people, the more precisely we can design personalised interventions.”

**What has surprised you the most while conducting your research?**

Before working on developing this method, we would separately design an experiment for either metabolomics or lipidomics. Interestingly, for metabolomics, we would already use a two-phase method. The reason for creating two phases here is to remove the lipids from the polar metabolites as they might disturb the metabolomics signal.

All this time, we have been discarding the apolar phase when extracting polar metabolites, which we now know can be very useful for lipidomic analyses. In hindsight, it is surprising how much useful material we've been discarding.

**Figure:**
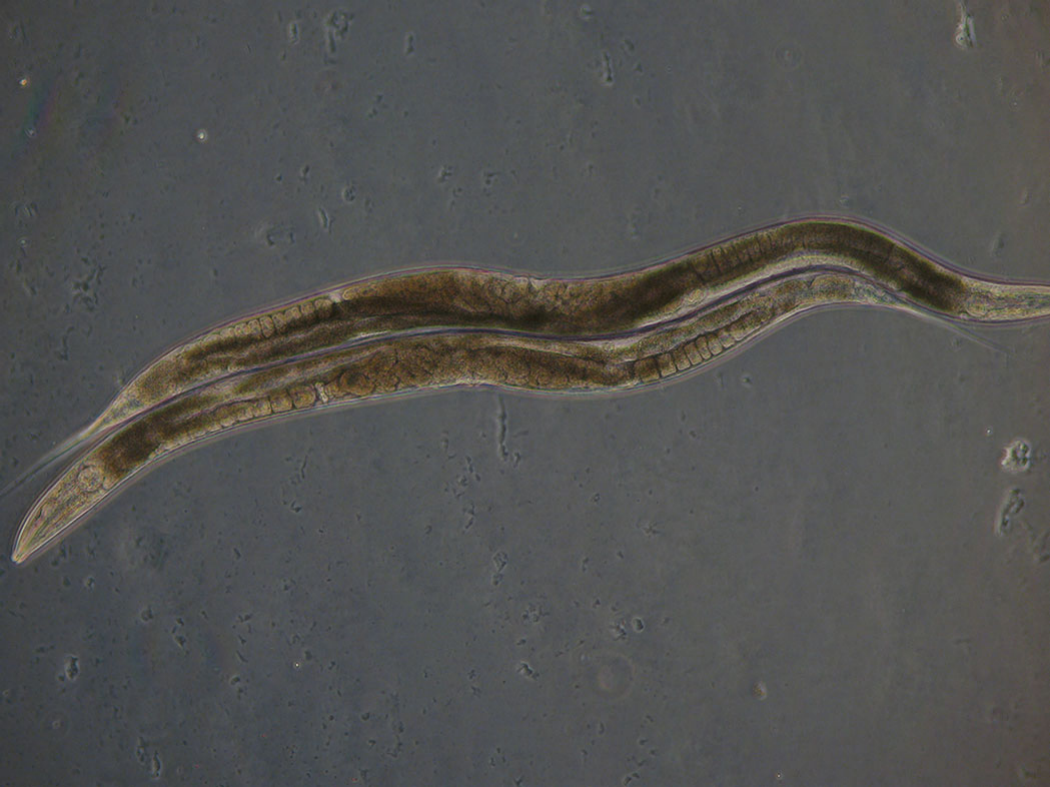
Two beautiful *C. elegans* hanging out together. Picture made in the Houtkooper group.

**Describe what you think is the most significant challenge impacting your research at this time and how will this be addressed over the next 10 years?**

It is clear that there are quite substantial differences between people, which, for instance, affects their sensitivity to drugs, diets and other interventions. Something that now still seems like a challenge is to really map the natural differences in metabolism between individuals. I think in 10 years we will have made huge steps towards personalised medicine. The better we understand and measure the differences in metabolism between people, the more precisely we can design personalised interventions.

“A competitive scientific community forces young scientists to perform result-oriented research, which is not beneficial to the spirit of science.”

**What changes do you think could improve the professional lives of early-career scientists?**

A competitive scientific community forces young scientists to perform result-oriented research, which is not beneficial to the spirit of science, moreover putting them under enormous pressure. Instead, if the community would evolve to be a more collaborative and co-operative enterprise, it would foster young scientists to acquire even more comprehensive knowledge on their research, thereby also producing impactful research.

**What's next for you?**

**MM:** This paper was part of my PhD research in which I focused on metabolism and ageing, which I completed in December 2020. I started my postdoctoral research in April 2021 in the group of Richard Possemato at New York University School of Medicine. Here, I will focus on cancer metabolism and will for sure make use of the method described in our paper.

**BS:** During the review process of this paper we were introduced to interesting work by others that, for instance, also performed omics on the protein pellet in a similar extraction. And, we've since acquired new mass spectrometers with additional capabilities. Combining these two things would lead to even more comprehensive methods.
